# Eyeballing stroke: Blood flow alterations in the eye and visual impairments following transient middle cerebral artery occlusion in adult rats

**DOI:** 10.1177/0963689720905805

**Published:** 2020-02-25

**Authors:** Jea-young Lee, Vanessa Castelli, Brooke Bonsack, Julián García-Sánchez, Chase Kingsbury, Hung Nguyen, Naoki Tajiri, Cesar V. Borlongan

**Affiliations:** 1 Center of Excellence for Aging and Brain Repair, Department of Neurosurgery and Brain Repair, University of South Florida College of Medicine, USA

**Keywords:** stroke, retinal ganglion cell, optic nerve, animal model, laser doppler

## Abstract

Middle cerebral artery occlusion in rodents remains a widely used model of ischemic stroke. Recently, we reported the occurrence of retinal ischemia in animals subjected to middle cerebral artery occlusion, owing in part to the circulatory juxtaposition of the ophthalmic artery to the middle cerebral artery. In this study, we examined the eye hemodynamics and visual deficits in middle cerebral artery occlusion-induced stroke rats. The brain and eye were evaluated by laser Doppler at baseline (prior to middle cerebral artery occlusion), during and after middle cerebral artery occlusion. Retinal function-relevant behavioral and histological outcomes were performed at 3 and 14 days post-middle cerebral artery occlusion. Laser Doppler revealed a typical reduction of at least 80% in the ipsilateral frontoparietal cortical area of the brain during middle cerebral artery occlusion compared to baseline, which returned to near-baseline levels during reperfusion. Retinal perfusion defects closely paralleled the timing of cerebral blood flow alterations in the acute stages of middle cerebral artery occlusion in adult rats, characterized by a significant blood flow defect in the ipsilateral eye with at least 90% reduction during middle cerebral artery occlusion compared to baseline, which was restored to near-baseline levels during reperfusion. Moreover, retinal ganglion cell density and optic nerve depth were significantly decreased in the ipsilateral eye. In addition, the stroke rats displayed eye closure. Behavioral performance in a light stimulus-mediated avoidance test was significantly impaired in middle cerebral artery occlusion rats compared to control animals. In view of visual deficits in stroke patients, closely monitoring of brain and retinal perfusion via laser Doppler measurements and examination of visual impairments may facilitate the diagnosis and the treatment of stroke, including retinal ischemia.

## Introduction

Stroke is ranked fourth among causes of death and is the leading cause of long-term disability in the United States^1–3^. Ischemic strokes comprise 87% of all strokes. In 2011, the direct and indirect total cost of stroke was 33.6 billion dollars and is projected to triple from 71.6 billion to 148.1 billion dollars between 2012 and 2030^3,4^. Ischemic stroke refers to a restriction of blood supply to a region or regions of the brain. As the blood supply is disrupted, oxygen and nutrition are not delivered to meet the metabolic demands, resulting in the development of an infarct area^5^. Retinal ischemia is a condition that occurs when blood is cut off from the retina, causing those cells to die. Because the retina is an extension of the central nervous system, physiological response to retinal ischemia is very similar to ischemic stroke^6^.

Middle cerebral artery occlusion (MCAO) in rodents is widely used as a model for ischemic stroke^7–11^. A filament is advanced through the internal carotid artery (ICA), blocking the base of the MCA^12,13^. Due to the proximity of the ophthalmic artery to the MCA, MCAO blocks both arteries, causing cerebral and retinal ischemia^14,15^. Previous reports have touched on retinal deficits arising from MCAO in rodents, which accompany neurostructural and neurological deficits^14,15^. The reported retinal dysfunctions include electroretinogram alterations^16–18^, retinal cell loss^14,15^, and retinal gliosis^18^. It has been proposed that a reduction in ocular blood flow, leading to ischemia-induced direct necrotic retinal cell death, and metabolic stress, resulting in apoptotic retinal cell death, are the degenerative pathways leading to these effects^14,15^. Clinically, ocular ischemia can occur in conjunction with cerebral stroke^19^ and, due to the retina’s high metabolic demand, any hindrance in the retina’s blood supply can easily lead to a reduced supply of oxygen, resulting in retinal ischemia^6,20^. Because retinal ischemia is a primary cause of blindness^6,20^, and with stroke a major cause of death and disability^21,22^, a better understanding of the underlying pathologies may help in developing novel treatment strategies. In this study, we evaluated the retinal hemodynamic effects of MCAO in rats, by using laser Doppler in concert with immunohistochemical and structural analysis of the optic nerve to further reveal the overlapping pathological deficits between retinal ischemia and MCAO.

## Materials and methods

### Subjects

All experiments were conducted in accordance with the National Institute of Health Guide and Use of Laboratory Animals, and were approved by the Institutional Animal Care and Use committee of the University of South Florida, Morsani College of Medicine, USA. Rats were housed two per cage in a temperature- and humidity-controlled room that was maintained on 12/12 hour-light/dark cycles. They had free access to food and water. All procedures were performed by personnel blinded to the treatment condition.

### Surgical procedures

Adult Sprague-Dawley rats (*n* = 24) were subjected to stroke (*n* = 16) or sham surgery (*n* = 8) anesthetized by a mixture of 1–2% isoflurane in nitrous oxide/oxygen (69%/30%) via face mask. Body temperature was maintained at 37 ± 0.3°C during the surgical procedures. The midline skin incision was made in the neck with subsequent exploration of the right common carotid artery (CCA), the external carotid artery, and ICA. A 4-0 monofilament nylon suture (27.0–28.0 mm) was advanced from the CCA bifurcation until it blocked the origin of the MCA. Animals were allowed to recover from anesthesia during MCAO. After 60 min of transient MCAO, animals were re-anesthetized with 1–2% isoflurane in nitrous oxide/oxygen (69%/30%) using a face mask and re-perfused by withdrawal of the nylon thread. Animals receiving the sham operation were anesthetized with 1–2% isoflurane in nitrous oxide/oxygen (69%/30%) via face mask. A midline incision was made in the neck and the right CCA was isolated, but without insertion of the monofilament into the CCA. The animals were then closed and allowed to recover from anesthesia. Brain and eye blood flow recordings were obtained using a laser Doppler (Perimed, Periflux System 5000, Las Vegas, NV, USA). For baseline brain, the laser Doppler probe was placed over the right frontoparietal cortical area supplied by the MCA. For baseline eye, the laser Doppler probe placed over the retina of the right eye. During MCAO, the brain decrease was reported as relative unit values, which were obtained by a laser Doppler probe placed again over the right frontoparietal cortical area. Similarly, during MCAO, the eye decrease was reported as relative unit values, which were obtained by a laser Doppler probe placed again over the retina of the right eye. The procedure mentioned above was repeated to generate the “after reperfusion MCAO” laser Doppler readings. During the operations, the body temperature was kept at 37°C with a feedback-controlled heating pad. Physiological outcome parameters of MCAO were kept constant across all animals throughout the experiment.

### Measurement of infarct volumes

Rats were euthanized with CO_2_ overdose and perfused with saline at 3 days (*n* = 9; sham = 4) and 14 days (*n* = 7; sham = 4) after MCAO for 2, 3, 5-triphenyltetrazolium chloride staining (TTC). The brains were quickly removed; seven coronal sections (one in the middle, two towards the back, and four towards the front) of 2-mm thickness were prepared^23^. The brain slices were incubated in a 2% solution of 2,3,5-triphenyltetrazolium chloride (Sigma Aldrich, St. Louis, MO, USA) with phosphate-buffered saline (PBS) at 37°C for 7 min in the dark and fixed in 4% buffered formaldehyde solution. The areas with normal cellular respiration stained dark red, while the infarct area remained unstained due to lack of cellular respiration. Brain slices were scanned, and infarct areas were measured using an imaging analysis software (Image J; National Institute of Health, Bethesda, MD). The infarct volumes were calculated with an edema correction.

### Enucleation and immunohistochemistry in the retina

The eyes were enucleated after a reference point was taken to label the superior pole and were immediately immersed in cold PBS (pH 7.4). The anterior segments of the eyes were removed and the retinas were isolated from the eyecup and post-fixed for 1 h in 4% glutaraldehyde in PBS. After being rinsed 3 x 10 min in PBS, retina tissues were processed as whole mounts. A monoclonal antibody against NeuN was obtained from Abcam (1:250; Cambridge, MA). It was processed free-floating in small vials with gentle agitation. Standard immunocytochemical techniques and immunocytochemical methods that have been described in detail in previous papers were used. For detection by immunofluorescence, the secondary antibodies were fluorescein conjugated anti-mouse IgG (1:500, Vector Lab., Burlingame, CA, USA). Immunofluorescence images were obtained on a Zeiss fluorescence microscope. As a control, some tissues were incubated in the same solution without the addition of the primary antibody. These control tissues showed no NeuN immunoreactivity. Retina sections were measured using an imaging analysis software (Image J; National Institute of Health, Bethesda, MD, USA). To compare the number of NeuN cells in the normal retina, and the ipsilateral and contralateral sides, we also counted labeled cells in five sequential fields, each 500 µm x 500 µm in area. Three tissue sections from each group (sham, contralateral, and ipsilateral) were measured.

### Measurement of the optic nerve

The optic nerves were collected at the same time point described above for structural analysis. The anterior segments of the eyes were removed and the optic nerve was isolated from the brain and post-fixed for 1 h in 4% glutaraldehyde in PBS. The optic nerves were stored in PBS until the time of analysis. Optic nerve images were obtained on an Olympus microscope. Optic nerve depths were measured using a microscope software. To determine the distributional types of optic nerve, we sampled fields from each group (three animals), each 500 µm x 500 µm in area. Analysis was done with a 40X Zeiss Plan-Apochromat objective.

### Eye score evaluation

After stroke, a behavioral evaluation for the eye tests was performed. The rats were evaluated for severity of stroke based on eye closure. The animals displayed a narrowing of the orbital area, a tightly closed eyelid, or an eye squeeze. An eye squeeze is defined as the contraction of the orbital muscles around the eye. A wrinkle may be visible around the eye. As a guideline, any eye closure that reduces the eye size by more than half was coded as “2”. We note that sleeping rats display closed eyes, and this may be mistaken for a tightly closed eyelid. Photographs of sleeping rats should therefore not be taken and/or coded. For each trial, animals received a score of 0, 1, 2, or 3 (0: normal; complete eye open, 1: mild; eye slightly closed, 2: moderate; eye is half closed 3: severe; complete eye closure).

### Avoidance task

This behavioral test followed our previous publications with modifications^7,24^. The training apparatus was a 50.5 × 16 × 27 cm box comprising a dark compartment made of black Plexiglass and a white compartment made of white Plexiglass. The ceiling of both compartments was transparent to allow observation of the animal’s activity. Both compartments had metal grid floors to which a continuous electric footshock (DC, 2 mA) could be delivered. Luminance at the white compartment of the box was 12.6 lux. Continuous white noise (70 dB) was broadcast through a speaker in the test room throughout the experimental session.

Each animal was placed individually in the box for 6 min habituation, during which it could freely explore the box. After the habituation period, the animal was placed in the dark compartment and the electric shock paired with white light (200 lux^25^) was immediately switched on in this compartment. This training continued until the subject stayed in the safe (i.e. white) compartment for 3 min. The retention test was undertaken on the following day. No electric shock was given, but the white light was switched on. The retention test started by placing the animal in the safe white compartment. If the rat entered the dark compartment within less than 3 min, the conditioning procedure described above was repeated. This training procedure continued until the animal stayed in the white compartment for 3 min. Following acquisition of the avoidance task, all subjects underwent the MCAO stroke or sham surgery (see separate Methods section on Surgical procedures). Thereafter, stroke and sham animals underwent shock/light recall tests at day 3 and day 14. Initially, animals were placed in the dark compartment, then shock was introduced, and the amount of time it took the animal to move to the white safe compartment was recorded. At 1 h after the shock recall test, the light recall test was conducted by placing the animal in the dark compartment, then introducing the white light, and the amount of time it took the animal to move to the white safe compartment was recorded. The subjects were removed from the box once they entered the white compartment.

### Statistical analysis

Data are presented as the mean ± SEM and analyzed using Sigma plot 8.0 program. Statview software was used to run analysis of variance followed by posthoc Bonferroni test. Statistical significance was preset at *p* < 0.05 (GraphPad version 5.01). The Kolmogorov-Smirnov test was performed to assess normality and the resulting values were less than 5% of the critical values.

## Results

### MCAO produces cerebral infarcts

**Figure 1.**
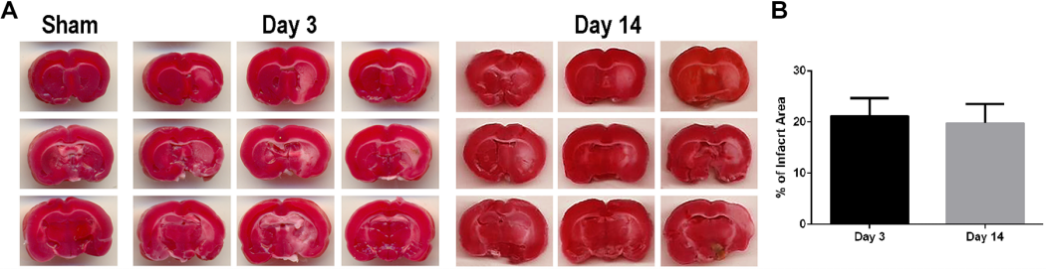
Middle cerebral artery occlusion (MCAO) produces typical cerebral infarcts. Tissue sections were processed with (2,3,5-triphenyltetrazolium chloride (TTC)) to reveal cerebral infarcts. (A) TTC-stained brain in sham and MCAO at 3 days and 14 days after stroke. The white color represents the infarct area. (B) Quantification of infarcts (percentage of impact area ± SEM) reveals visible infarcts at 3 days and 14 days after stroke.

As routinely reported, the 60-min MCAO stroke produced consistent cortical and striatal infarcts within the ipsilateral hemisphere as revealed by TTC staining (Figure 1). The infarct size average was 19.8% at 3 days, and 21.21% at 14 days after stroke compared to sham animals (*p* < 0.05).

### MCAO induces ganglion cell death in the retina

**Figure 2.**
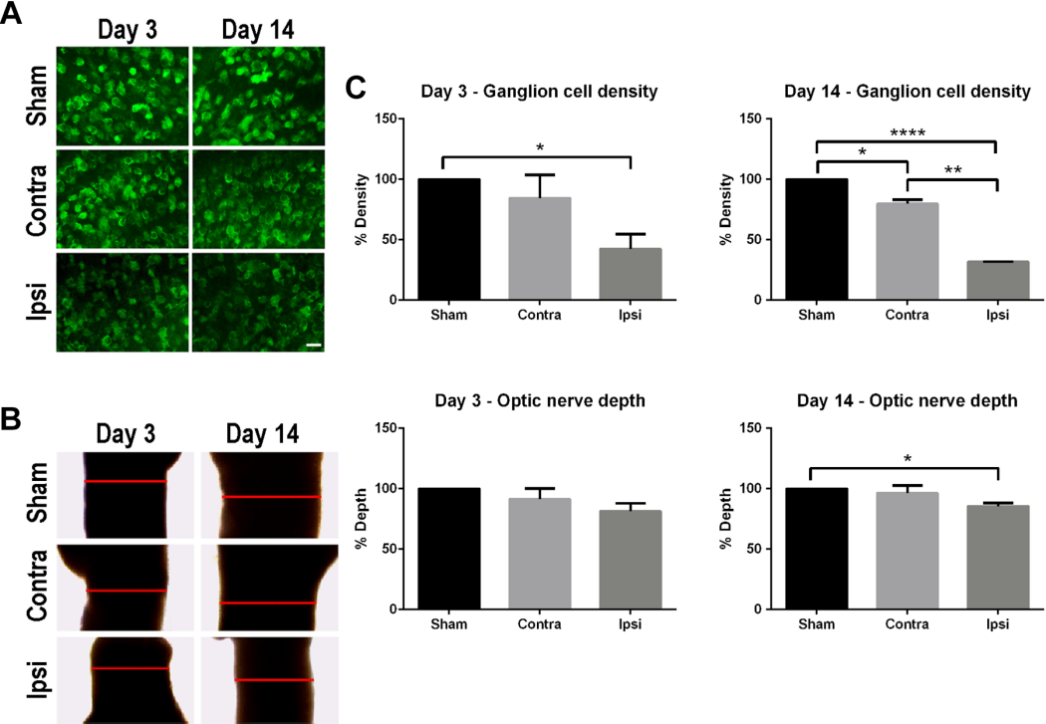
Middle cerebral artery occlusion (MCAO) alters retina cell survival and optic nerve depth. Representative images of NeuN immunohistochemistry in retina to reveal retinal ganglion cell density (A; scale bar = 25 um) and optic nerve depth (B; red line), which were subsequently quantified and analyzed (C). The depth of the optic nerve and the density of retinal ganglion cells in MCAO rats showed a general reduction compared to sham at 3 days and 14 days after stroke. Significance: *p < 0.05; **p < 0.01; ***p < 0.001.

There was a reduction of 57.66% (*p* < 0.05) in ganglion cell density in the ipsilateral retina of the MCAO animals compared to sham animals at 3 days after stroke (Figures 2(a) and (c)). This reduction exacerbated further to 68.21% (*p* = 0.000004) at 14 days after stroke. On the contralateral side, the ganglion cell density decreased to 13.55% (*p* = 0.27) and 20.41% (*p* = 0.009) compared to sham animals at 3 and 14 days after surgery, respectively. The differences between the contralateral side and the ipsilateral side in ganglion cell density were 44.11% (*p* = 0.015) and 47.91% (*p* = 0.0014) at 3 and 14 days post-surgery, respectively.

### MCAO decreases optic nerve length (depth)

In general, there was a significant reduction in the depth of the ipsilateral optic nerve compared to sham group (Figures 2(b) and (c)). At 3 days after MCAO, the depth of the ipsilateral and contralateral optic nerves decreased by 8.62% (*p* = 0.23) and 18.56% (*p* = 0.12), respectively, compared to sham (*p* < 0.05). When the ipsilateral optic nerve was compared to the contralateral optic nerve, there was a 9.93% reduction that almost reached significance (*p* = 0.054). At 14 days after MCAO, the depths of optic nerves decreased by 3.69% in the contralateral (*p* = 0.42), whereas it was significantly reduced by 14.5% in the ipsilateral (*p* = 0.01) compared to sham groups. Another trend of reduction by 10.8% (*p* = 0.075) was detected in the ipsilateral optic nerve depth when compared to the contralateral side.

### MCAO reduces blood flow to both the brain and the eye

**Figure 3.**
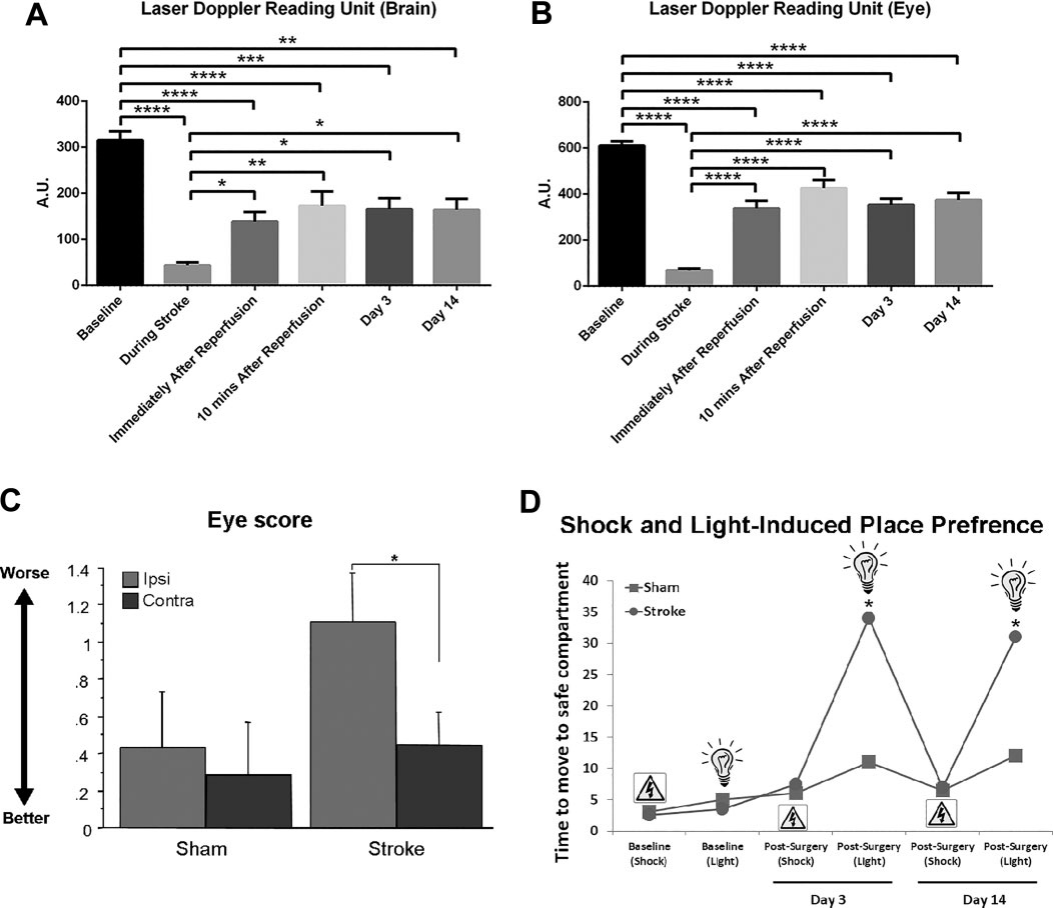
Middle cerebral artery occlusion (MCAO) reduces blood flow in the brain and the eye and induces visual impairments. The laser Doppler readings in both brain (A) and eye (B) displayed a similar pattern, characterized by significant blood flow reduction during MCAO, then recovery towards baseline levels after reperfusion. Evaluation of ipsilateral eye closure revealed that stroke animals displayed a higher score (i.e. a half-closed eye) compared to sham group with only very slight or no detectable eye closure (C). Moreover, the stroke animals exhibited visual impairments as revealed in an avoidance test (D). Prior to stroke surgery, both groups of animals learned the avoidance task by successfully preferring the safe place when the electric shock or the light stimulus was presented. After the stroke surgery, the MCAO animals continued to prefer the safe place to avoid the electric shock, but they failed this task when presented only with the light stimulus compared to sham animals, at 3 and 14 days post-surgery. Significance: *p < 0.05.

Laser Doppler readings at baseline, during the MCAO stroke surgery, and 3 and 14 days after stroke revealed altered blood flow in both the brain and the eye (Figures 3(a) and 3(b)). Baseline laser Doppler blood flow perfusion units of brain and eye were 315 ± 19 and 610 ± 18, respectively. During MCAO, there were significant reductions in blood flow to both the brain and the eye. The average of laser Doppler readings for the brain and the eye were 44.48 ± 16.73 and 49.77 ± 27.21, which equated to about 80% and 89% reductions respectively, compared to baseline readings in stroke animals. The laser Doppler readings increased after reperfusion and trended towards near baseline levels by 14 days after stroke (**p < 0.01; ***p < 0.001; ****p < 0.0001).

### Eye score test

After stroke, the animals were evaluated for severity of stroke based on eye closure. Stroke animals showed a significantly higher score of eye closure compared to sham group (Figure 3(c)). The average score for stroke animals was around 1.1 thus indicating a half-closed eye compared to around 0.4, indicating no detectable to very mild eye closure in sham animals (*p* < 0.05).

### Avoidance test

Before and after surgery, the animals were subjected to the avoidance test (Figure 3(d)). Prior to stroke and sham surgeries, both groups of animals learned the avoidance task by successfully preferring the safe place when the electric shock was introduced or when presented with the light stimulus alone that was previously associated with the electric shock. Within 5 seconds of electric shock or light stimulus introduction, the animals moved to the safe compartment. However, following the stroke surgery, although the MCAO animals continued to prefer the safe place to avoid the electric shock, they were not able to retain such tasks when presented with the light stimulus compared to sham animals, at 3 and 14 days post-surgery. Collectively, these results indicated that although memory retention of the safe place was intact (albeit in the presence of electric shock), the animals were not able to process the “light stimulus” visual cue, likely due to retinal damage associated with stroke (*p* < 0.05).

## Discussion

This study reports that filament MCAO model results in retinal ischemia, causing damage to the retinal ganglion cell and the optic nerve^14,15,18^. A similar pattern of alterations in blood flow were detected both the brain and the eye prior to stroke, during MCAO, and at 3 days and 14 days after stroke. The histopathological and haemodynamics profile of MCAO-induced retinal ischemia produced functional correlates as evidenced by visual impairments in a simple cognitive task. That retinal ischemia is demonstrated here to closely accompany the MCAO stroke model replicates the clinical setting with equally high incidence of visual impairments in ischemic stroke patients. The observation of retinal ischemia with unfavorable haemodynamics, histopathological, and behavioral manifestations warrants its recognition as a major pathological consequence of stroke.

The occurrence of retinal ischemia after MCAO-induced ischemic stroke is likely due to the proximity of the ophthalmic artery to the MCA, in that occlusion of the MCA is almost certain to block blood supply to the ophthalmic artery. Moreover, the retina is heavily vascularized and thus highly susceptible to vascular metabolic stress. Laser Doppler readings in the retina closely paralleled the brain cerebral blood flow perfusion, characterized by significant reduction in blood flow during MCAO, which reverted to baseline after reperfusion. Our study showed the retinal damage worsened at 14 days after MCAO, extending the alterations seen in the acute phase from previous reports^14,15,26^.

The MCAO-induced retinal ischemia may trigger a cascade of cell death events, most mitochondrial dysfunctions, as we recently reported^27^. At the functional level, stroke animals displayed eye closure that may affect their perception and learning of visual cues. Indeed, stroke animals were significantly impaired in their performance of the passive avoidance task when light stimulus was used to alert an impending aversive condition. Some functional deficits have been reported with the retinal ischemia induced by MCAO, such as electroretinogram alterations^16–18^, retinal cell loss^14,15^, and retinal gliosis^18^.

A simple observation but with far-ranging application of laser Doppler that we discovered here is its extended use for detecting blood flow in eye. We showed here that the laser Doppler is a sensitive tool in monitoring blood flow, thus it stands as a reliable alternative method to its current use for intra-cranial regional cerebral blood flow recording for measuring the haemodynamics of MCAO. Laser Doppler reading in the eye is less invasive and does not require additional invasive procedures to the head, thereby circumventing trauma to the already injured stroke brain. Accordingly, we advance the novel use of laser Doppler of the eye as an approach to reduce the chance of trauma and infection to the head, allowing a minimally invasive approach to the detection of altered blood flow after stroke. We envision a rapid assessment of the severity of stroke using a human-compatible laser Doppler in the emergency medicine settings that would facilitate early initiation of treatment intervention, such as tissue plasminogen activator (tPA), which is limited to 4.5 hours after stroke onset.

Although further studies are required to fully reveal the direct correlation between the pathological deficits arising from retinal ischemia and the neurobehavioral outcomes^7^, the current finding raises an important question about some commonly used behavioral tests that rely on visual cues on MCAO subjects^28^. The change in behavioral outcomes observed in those tests might be due to the visual impairments, the stroke brain, or a combination of both. If the results are caused by a visual impairment and not a brain deficit, they can undermine the validity of the tests. For example, the Morris Water Maze, a commonly used cognitive test, requires animals to use visual cues in conjunction with memory to find the hidden platform. The poor performance of the animals might be due to the inability to visualize the environmental cues to navigate to the platform, and not necessarily a cause-and-effect outcome of the stroke brain. Hence, the result of the test might not indicate the direct correlation between brain damage and functional deficit. Proper design of memory and learning paradigms that incorporate visual perception may allow delineation of true cognitive deficits from visual impairments. In the present study, we were able to detect that memory retention of the passive avoidance was intact, yet the visual perception of light stimulus was impaired in stroke animals.

Visual impairments after stroke have been reported in clinical settings^29–31^. Approximately 30% of stroke patients experience some visual impairments^31^. Hence, we suggest that stroke patients should also be screened for retinal damage as part of the disease differential diagnosis and its treatment regimen to improve the overall clinical outcome. Clinical outcomes from transplantation of stem cells in stroke, although mostly focused on repairing the brain^32–40^, may benefit from exploring the grafted cells deposition and function in the eye^41,42^.

In conclusion, the present study characterized the functional correlates of retinal ischemia in the MCAO rat model, in particular demonstrating the changes in haemodynamics in the eye that closely resembled the cerebral blood flow alterations as measured by laser Doppler. Histopathological deficits, as evidenced by retinal ganglion cell and optic nerve damage, and visual impairments, as revealed by failure to recognize light stimulus-cued avoidance test, accompanied the MCAO-induced retinal ischemia. A better understanding of stroke and retinal ischemia pathophysiology can help to further understand the disease and to improve the current standard treatment of care for stroke patients. To this end, enhancing the retinal ganglion cell survival, such as by stem-cell transplantation to repair mitochondrial dysfunction^27^, may prove to be of therapeutic value to stroke and retinal ischemia.
